# Reverse total shoulder arthroplasty versus open reduction internal fixation for proximal humerus fractures in elderly patients: an age-stratified propensity score–matched analysis

**DOI:** 10.1016/j.jsea.2026.100064

**Published:** 2026-07-17

**Authors:** Muaaz Wajahath, Jawad Saad, Magd Boutany, Ameen Suhrawardy, David Abdelnour, Abdul Lateef Shafau, Alqasim Elnaggar, Rahul Vaidya

**Affiliations:** a Michigan State University College of Human Medicine, East Lansing, MI, USA; b Wayne State University School of Medicine, Detroit, MI, USA; c Department of Orthopaedic Surgery, Detroit Medical Center, Detroit, MI, USA

**Keywords:** Proximal humerus fracture, Reverse total shoulder arthroplasty, Open reduction internal fixation, Elderly, Propensity score matching, Outcomes

## Abstract

**Background:**

The optimal surgical management of displaced proximal humerus fractures in elderly patients remains controversial. Reverse total shoulder arthroplasty (rTSA) has gained increasing acceptance for complex fracture patterns, yet comparative data against open reduction and internal fixation (ORIF) across age strata are limited. This study compared reoperation rates, emergency department (ED) utilization, and dislocation following rTSA versus ORIF in propensity score–matched cohorts stratified by age.

**Methods:**

Using the TriNetX multi-institutional research network, patients aged 65-74 years and 75-84 years who underwent rTSA or ORIF for proximal humerus fractures were identified. Propensity score matching was performed using nearest-neighbor matching with a 0.150 standard deviation caliper. The primary outcome was coded shoulder-related return to surgery at 1 and 2 years. Secondary outcomes included all-cause ED utilization and coded dislocation when available.

**Results:**

After matching, 748 patients (374 per group) were analyzed in the 65 to 74-year cohort and 882 patients (441 per group) in the 75 to 84-year cohort. Patients undergoing rTSA had significantly more 4-part fractures (34.2% vs. 6.4% in the 65 to 74-year cohort, *P* < .001). At 2-year follow-up, reoperation rates did not differ significantly in either age group (65-74 years: rTSA 5.62% vs. ORIF 3.48%, risk ratio 1.62, *P* = .16; 75-84: rTSA 4.54% vs. ORIF 5.67%, risk ratio 0.80, *P* = .444). Kaplan-Meier survival analysis confirmed no significant differences in freedom from surgical failure. ED utilization did not differ significantly between groups, and dislocation was comparable in the 75 to 84-year cohort for which dislocation data were available.

**Conclusion:**

In propensity score–matched cohorts, rTSA and ORIF demonstrated comparable coded reoperation rates and all-cause ED utilization at 1- and 2-year follow-up. These findings persisted despite rTSA patients having more complex fracture patterns; however, because fracture morphology and patient-reported outcomes were not directly matched or captured, the results should be interpreted as hypothesis-generating real-world associations rather than evidence of treatment superiority.

Proximal humerus fractures are among the most common fragility fractures in older adults and continue to impose a substantial clinical burden in an aging population. Over the past decade, operative management patterns have shifted considerably: open reduction and internal fixation (ORIF) remains widely used, but reverse total shoulder arthroplasty (rTSA) has expanded rapidly, while hemiarthroplasty has steadily fallen out of favor. Recent national database studies suggest that this shift has been accompanied not only by changes in implant selection, but also by growing interest in downstream utilization and readmission outcomes after treatment for proximal humerus fracture.^3^^,^^11^

Treatment selection for displaced proximal humerus fractures in elderly patients remains challenging because the decision extends beyond fracture displacement alone. Bone quality, fracture morphology, tuberosity integrity, rotator cuff status, medical frailty, rehabilitation tolerance, and baseline functional demand all influence whether a reconstructive strategy or an arthroplasty-based strategy is most appropriate. Contemporary treatment algorithms have therefore emphasized individualized decision-making grounded in patient biology and fracture complexity rather than a uniform age cutoff alone.^19^

ORIF with locking plate technology continues to offer the theoretical advantages of anatomic reduction, preservation of the native joint, and avoidance of prosthetic complications. However, these benefits may be offset in older patients by osteoporotic bone, medial comminution, varus collapse, screw cutout, fixation failure, and reoperation. In 1 modern series, locking plate fixation in patients older than 60 years remained associated with a high complication burden, reinforcing persistent concerns regarding durability in elderly fracture patterns.^2^

In parallel, rTSA has become increasingly attractive for complex 3-part and 4-part fractures because it is less dependent on tuberosity healing and rotator cuff function for restoration of shoulder elevation. High-level comparative studies have strengthened the rationale for its use. The DelPhi randomized trial demonstrated superior 2-year outcomes for rTSA compared with plate fixation in displaced proximal humeral fractures in elderly patients, and a multicenter randomized trial similarly showed that rTSA provided better shoulder function than hemiarthroplasty in patients aged 70 years or older.^5^^,^^10^

Still, the comparative literature remains nuanced rather than uniform. Meta-analyses and systematic reviews have generally favored rTSA for forward flexion, patient-reported outcomes, and lower revision risk, but they have also shown that ORIF may retain advantages in external rotation and that complication profiles depend heavily on which end point is examined. Importantly, much of the existing literature has focused on pooled elderly cohorts with complex 3-part and 4-part fractures, leaving the effect of narrower age strata on comparative durability less well defined.^4^^,^^16^^,^^20^

Accordingly, the purpose of the present study was to compare coded reoperation, emergency department (ED) utilization, and dislocation after rTSA versus ORIF for proximal humerus fractures in 2 clinically relevant elderly age groups: 65 to 74 years and 75 to 84 years. By isolating these adjacent but biologically distinct age cohorts within a large multi-institutional database, this study sought to describe whether the relative balance between fixation and arthroplasty differed with advancing age in real-world practice. We hypothesized that rTSA would demonstrate comparable coded surgical durability to ORIF, while recognizing that administrative data cannot establish treatment superiority specific to the fracture pattern.

## Materials and methods

### Study design and data source

We performed a retrospective cohort study using the TriNetX Research Network, a federated database of de-identified electronic health record data from participating academic and community health systems. TriNetX aggregates longitudinal patient information including demographics, diagnoses, procedures, laboratory results, and medication records. Diagnoses and procedures are captured using standardized coding systems, including the International Classification of Diseases, 10th Revision (ICD-10) and Current Procedural Terminology. This study was reported in accordance with the REporting of studies Conducted using Observational Routinely-collected health Data statement.^21^ Because the TriNetX platform contains only de-identified data, this study was considered exempt from institutional review board oversight and informed consent requirements.

### Patient selection

Patients with a diagnosis of proximal humerus fracture (ICD-10 S42.2xx) who subsequently underwent either rTSA or ORIF were identified within TriNetX. The date of the index surgical procedure was defined as the date of cohort entry. To evaluate the potential influence of age on comparative treatment outcomes, patients were stratified into 2 prespecified age groups: 65 to 74 years and 75 to 84 years. Patients younger than 65 years (n = 65,819) and older than 84 years (n = 2,069) in the initial proximal humerus fracture query were not included in the primary age-stratified analysis. An exploratory cohort of patients aged 85 years or older was queried initially; however, treatment-specific matched event counts were too small to support meaningful matched comparative analysis, and that cohort was excluded from the primary study.

### Covariates and propensity score matching

To reduce baseline differences between treatment groups, 1:1 propensity score matching was performed separately within each age stratum using nearest-neighbor matching with a caliper width of 0.150 standard deviations. Patients were matched without replacement so that each patient contributed only once to the matched analysis. Covariates entered into the propensity model included age, sex, race, ethnicity, obesity, diabetes mellitus, osteoporosis, chronic obstructive pulmonary disease, chronic kidney disease, heart failure, nicotine dependence, primary shoulder osteoarthritis, and rotator cuff pathology. No manual imputation was performed for missing covariate data. Procedure year, treating institution, and surgeon-level identifiers were not included in the matching model because they were not available in the de-identified aggregate query output used for this study.

Covariate balance after matching was assessed using standardized mean differences (SMDs), with an SMD of <0.10 considered indicative of adequate balance. Fracture morphology, including 2-part, 3-part, and 4-part fracture designations based on ICD-10 coding, was analyzed descriptively but was intentionally not included in the matching algorithm. This decision was made because fracture complexity is a major determinant of treatment selection in clinical practice; however, it also means that the matched comparisons should be interpreted as real-world associations rather than causal estimates matched by fracture pattern.

### Outcomes

The primary outcome was coded shoulder-related return to surgery, defined as any subsequent shoulder-related operative procedure code after the index procedure within the TriNetX query output. This end point was intended to capture coded operative revision or reoperation events, including shoulder arthroplasty/revision or fixation-related return to the operating room when recorded through standardized procedure coding. It was not designed to adjudicate radiographic failure, minor nonoperative events, or the specific operative indication from clinical notes. Primary outcomes were evaluated at 1 and 2 years after surgery.

Secondary outcomes included ED utilization and dislocation. ED utilization was captured as any all-cause ED encounter within 1 year after the index procedure and was interpreted as a general health-care utilization marker rather than a shoulder-specific complication. Dislocation was evaluated when available from the query output and should be interpreted as a coded dislocation or instability-related diagnosis rather than a manually adjudicated glenohumeral instability event. Secondary outcomes were assessed at 1-year follow-up, with 2-year survival analyses additionally performed for the primary end point of surgical failure.

### Statistical analysis

Categorical variables were summarized as counts and percentages, and continuous variables as means with standard deviations. Between-group comparisons were performed using the chi-square test or Fisher exact test, as appropriate. Risk differences, risk ratios (RRs), odds ratios, and 95% confidence intervals (CIs) were calculated for binary outcomes.

Time-to-event analyses for surgical failure were performed using Kaplan-Meier methods, with comparisons between groups assessed using the log-rank test. Cox proportional hazards regression was used to estimate hazard ratios (HRs) with 95% CIs. The proportional hazards assumption was evaluated within the TriNetX platform using Schoenfeld residual testing. A 2-sided *P* value of <0.05 was considered statistically significant. Because multiple outcomes were evaluated across age strata, *P* values were interpreted descriptively and no formal correction for multiple comparisons was applied. All analyses were performed within the TriNetX analytics environment.

## Results

### Matched cohorts and baseline balance

**Figure 1:**
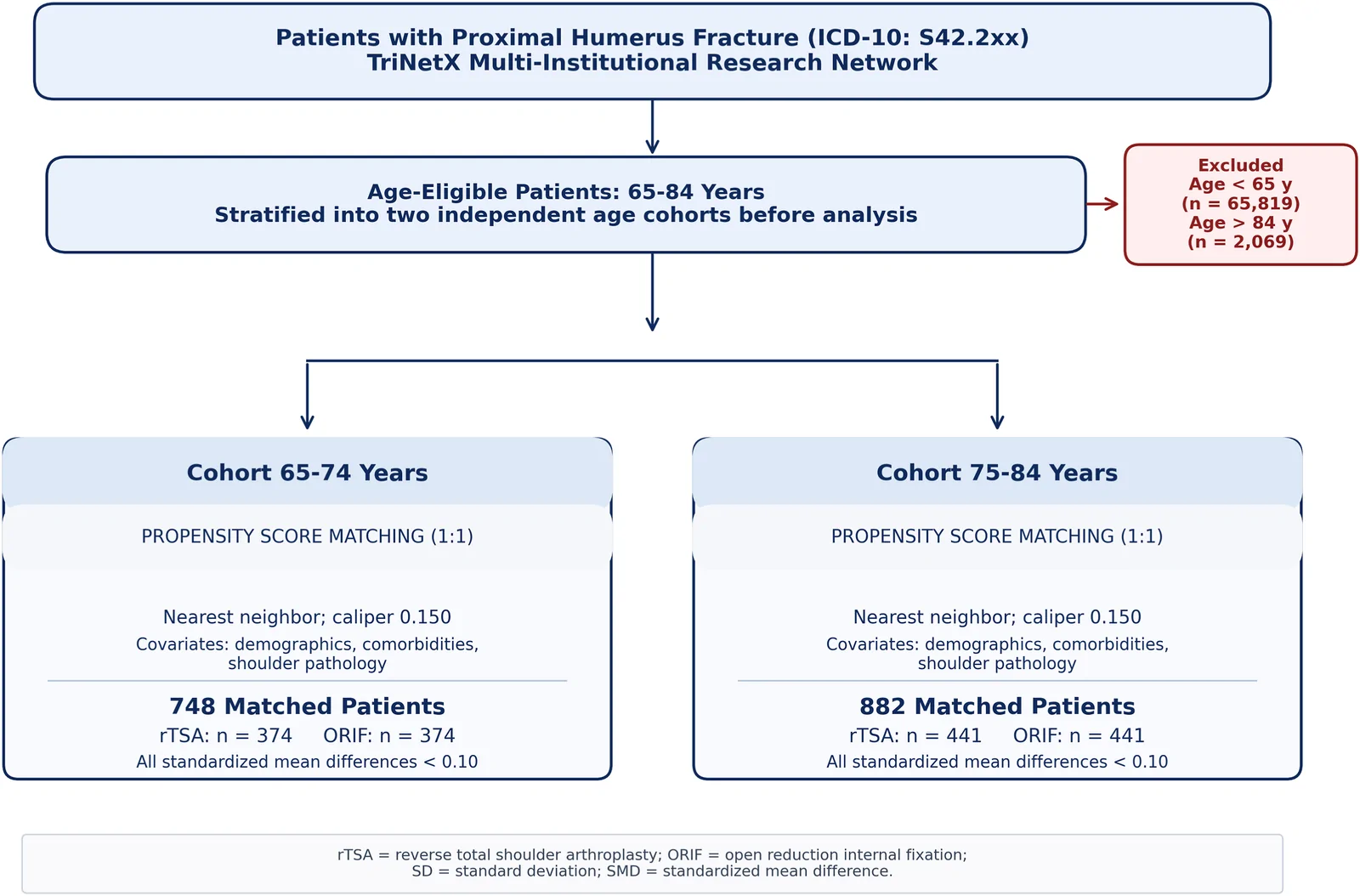
Cohort selection flow diagram. Patients with proximal humerus fracture (ICD-10: S42.2xx) who underwent rTSA or ORIF were identified from the TriNetX Research Network and stratified by age into 2 independent cohorts (65-74 yr; 75-84 yr). Each cohort underwent 1:1 nearest-neighbor propensity score matching (caliper, 0.150 SD) on demographic and clinical covariates. Final matched cohorts comprised 748 patients (Cohort A) and 882 patients (Cohort B); all standardized mean differences were <0.10. *rTSA*, reverse total shoulder arthroplasty; *ORIF*, open reduction and internal fixation; *SD*, standard deviation; *SMD*, standardized mean difference.

After 1:1 propensity score matching, the 65 to 74-year cohort included 748 patients, with 374 patients in each treatment group. The 75 to 84-year cohort included 882 patients, with 441 patients in each treatment group (Fig. 1).

**Figure 2:**
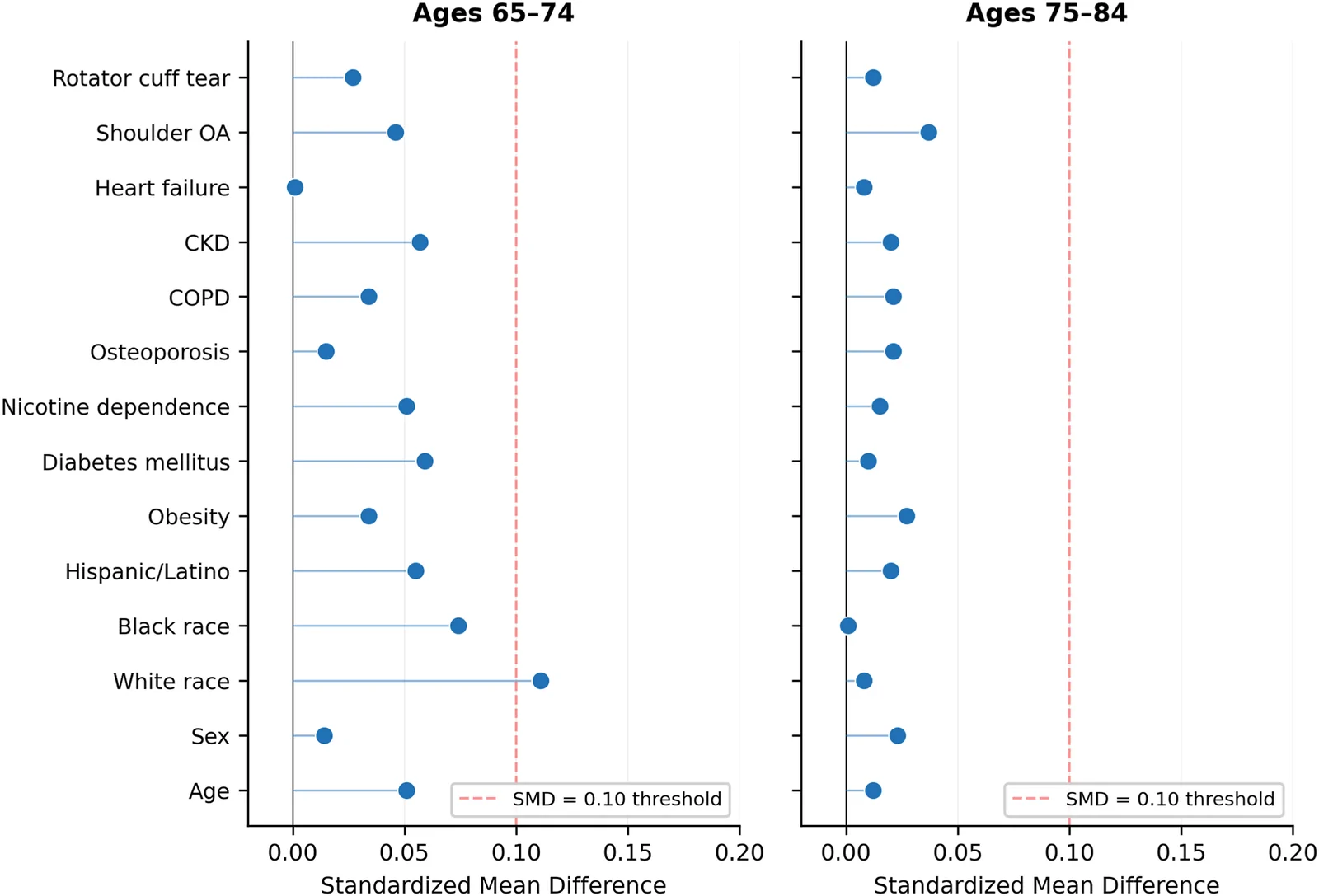
Covariate balance after propensity score matching. Love plots of absolute standardized mean differences for all matched covariates in Cohort A (*Left*) and Cohort B (*Right*) after 1:1 matching. Dashed red line marks the balance threshold (SMD = 0.10). All covariates achieved adequate balance in both cohorts. *SMD*, standardized mean difference; *OA*, osteoarthritis; *CKD*, chronic kidney disease; *COPD*, chronic obstructive pulmonary disease.

Postmatch covariate balance was excellent in both age strata. All matched covariates demonstrated SMDs below 0.10, and most were below 0.05, indicating minimal residual imbalance between the rTSA and ORIF groups (Tables I and II; Fig. 2). Across both cohorts, the matched populations were predominantly female and White, and the prevalence of major comorbidities, including diabetes mellitus, osteoporosis, chronic kidney disease, chronic obstructive pulmonary disease, heart failure, nicotine dependence, and rotator cuff pathology, was similar between treatment groups. Fracture morphology was not included in the propensity score model and was therefore analyzed descriptively as a marker of real-world treatment selection.

**Table I:** Baseline demographics after propensity score matching: ages 65-74 year.

Characteristic	rTSA (n = 374)	ORIF (n = 374)	*P* value	SMD
Age, mean ± SD	64.9 ± 3.39	65.1 ± 3.45	.487	0.051
Female sex	309 (82.6%)	311 (83.2%)	.846	0.014
White race	340 (90.9%)	351 (93.9%)	.130	0.111
Black or African American	15 (4.0%)	10 (2.7%)	.309	0.074
Hispanic or Latino	13 (3.5%)	17 (4.5%)	.456	0.055
Obesity	127 (34.0%)	121 (32.4%)	.641	0.034
Diabetes mellitus	113 (30.2%)	103 (27.5%)	.420	0.059
Nicotine dependence	65 (17.4%)	58 (15.5%)	.490	0.051
Osteoporosis	55 (14.7%)	53 (14.2%)	.835	0.015
COPD	44 (11.8%)	40 (10.7%)	.643	0.034
Chronic kidney disease	35 (9.4%)	29 (7.8%)	.433	0.057
Heart failure	33 (8.8%)	33 (8.8%)	1.000	<0.001
Shoulder osteoarthritis	33 (8.8%)	38 (10.2%)	.533	0.046
Rotator cuff tear	16 (4.3%)	14 (3.7%)	.709	0.027

*SD*, standard deviation; *rTSA*, reverse total shoulder arthroplasty; *SMD*, standardized mean difference; *COPD*, chronic obstructive pulmonary disease; *ORIF*, open reduction and internal fixation.

**Table II:** Baseline demographics after propensity score matching: ages 75-84 year.

Characteristic	rTSA (n = 441)	ORIF (n = 441)	*P* value	SMD
Age, mean ± SD	73.8 ± 3.54	73.8 ± 3.59	.858	0.012
Female sex	361 (81.9%)	357 (81.0%)	.729	0.023
White race	395 (89.6%)	396 (89.8%)	.912	0.008
Black or African American	10 (2.3%)	10 (2.3%)	1.000	<0.001
Hispanic or Latino	23 (5.2%)	25 (5.7%)	.767	0.020
Diabetes mellitus	123 (27.9%)	125 (28.3%)	.881	0.010
Osteoporosis	107 (24.3%)	103 (23.4%)	.752	0.021
Overweight/obesity	95 (21.5%)	100 (22.7%)	.685	0.027
Chronic kidney disease	59 (13.4%)	56 (12.7%)	.764	0.020
COPD	52 (11.8%)	49 (11.1%)	.751	0.021
Heart failure	45 (10.2%)	46 (10.4%)	.912	0.008
Nicotine dependence	44 (10.0%)	46 (10.4%)	.824	0.015
Shoulder osteoarthritis	49 (11.1%)	44 (10.0%)	.584	0.037
Rotator cuff tear	15 (3.4%)	16 (3.6%)	.855	0.012

*SD*, standard deviation; *ORIF*, open reduction and internal fixation; *rTSA*, reverse total shoulder arthroplasty; *SMD*, standardized mean difference; *COPD*, chronic obstructive pulmonary disease.

### Fracture pattern distribution

**Figure 3:**
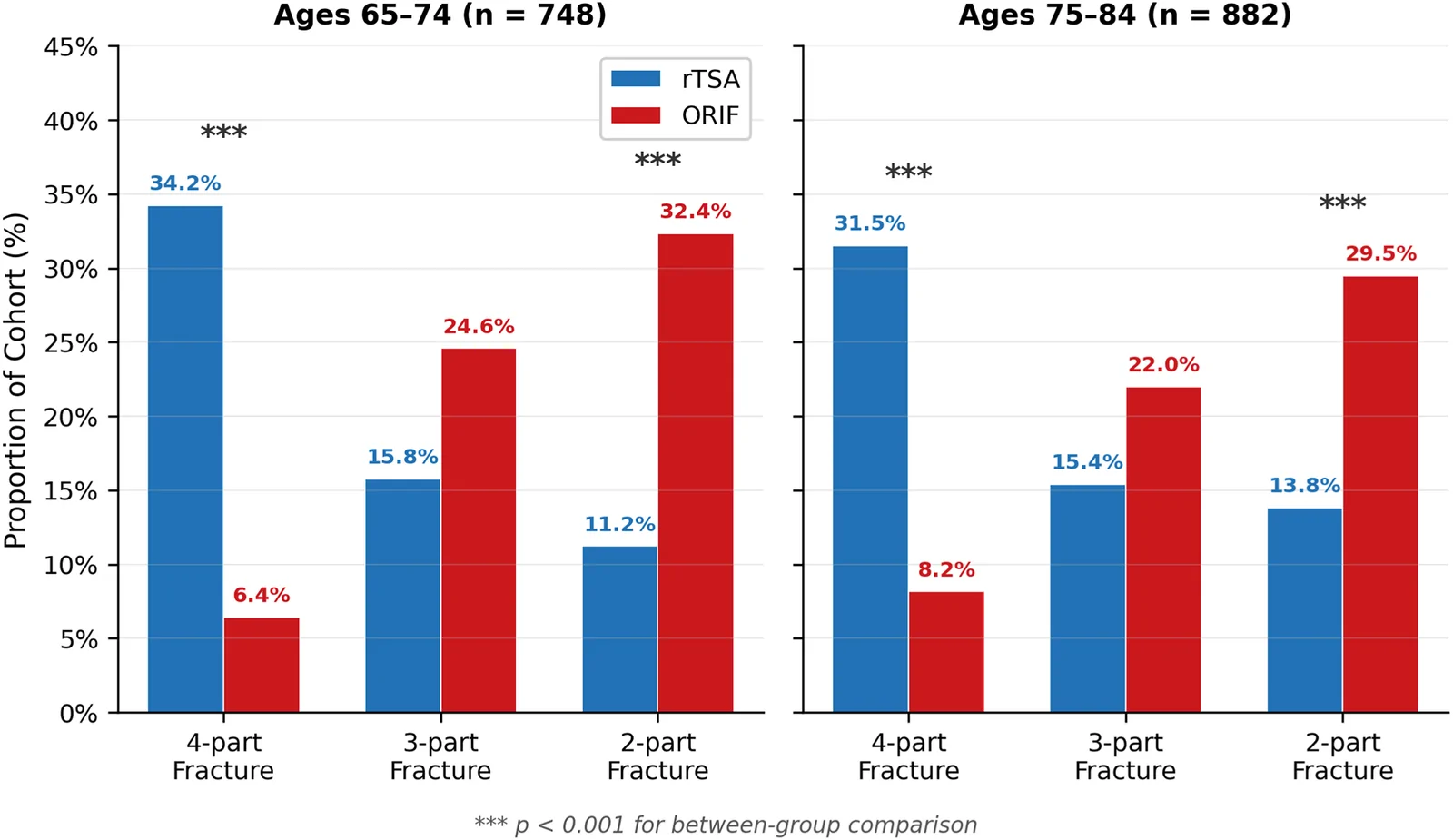
Fracture morphology distribution by treatment group. Proportion of 2-part, 3-part, and 4-part fractures by treatment group in each age cohort prior to propensity score matching. rTSA patients had significantly higher rates of 4-part fractures in both cohorts (*P* < .001), consistent with expected clinical selection patterns. Fracture morphology was excluded from the matching model. ∗∗∗*P* < .001 by chi-square test. *rTSA*, reverse total shoulder arthroplasty; *ORIF*, open reduction and internal fixation.

Fracture morphology remained markedly different between treatment groups and reflected expected treatment-selection patterns. In both age strata, patients treated with rTSA had substantially higher proportions of 4-part fractures, whereas patients treated with ORIF had higher proportions of 2-part fractures (Table III; Fig. 3).

**Table III:** Fracture morphology by treatment cohort (not matched).

Fracture pattern	Ages 65-74 yr	ORIF	P	Ages 75-84 yr	ORIF	*P* value
	rTSA			rTSA		
4-part, R	74 (19.8%)	14 (3.7%)	**<.001**	83 (18.8%)	19 (4.3%)	**<.001**
4-part, L	54 (14.4%)	10 (2.7%)	**<.001**	56 (12.7%)	17 (3.9%)	**<.001**
3-part, R	30 (8.0%)	43 (11.5%)	.109	39 (8.8%)	51 (11.6%)	.182
3-part, L	29 (7.8%)	49 (13.1%)	**.017**	29 (6.6%)	46 (10.4%)	**.040**
2-part, R	25 (6.7%)	57 (15.2%)	**<.001**	39 (8.8%)	60 (13.6%)	**.025**
2-part, L	17 (4.5%)	64 (17.1%)	**<.001**	22 (5.0%)	70 (15.9%)	**<.001**

*ORIF*, open reduction and internal fixation; *rTSA*, reverse total shoulder arthroplasty; *R*, right; *L*, left.

Bold *P* values indicate statistical significance. Fracture morphology was not included in the propensity score matching model.

In the 65 to 74-year cohort, overall 4-part fracture coding was more common in the rTSA group than in the ORIF group (34.2% vs. 6.4%, *P* < .001), while overall 2-part fracture coding was more common in the ORIF group (32.3% vs. 11.2%, *P* < .001). Three-part fracture coding was more evenly distributed, although residual differences remained in some coded subcategories.

A similar pattern was observed in the 75 to 84-year cohort. Overall 4-part fracture coding was more common in the rTSA group than in the ORIF group (31.5% vs. 8.2%, *P* < .001), whereas overall 2-part fracture coding was more common in the ORIF group (29.5% vs. 13.8%, *P* < .001). These findings confirm that rTSA was preferentially used for more complex coded fracture patterns despite the otherwise well-balanced matched cohorts.

### Primary outcome: surgical failure/reoperation

#### One-year outcomes

In the 65 to 74-year cohort, surgical failure within 1 year occurred in 18 rTSA patients (4.81%) and 12 ORIF patients (3.21%) (RR, 1.50; 95% CI, 0.73-3.07; *P* = .264) (Table IV). Kaplan-Meier analysis showed no significant difference in freedom from surgical failure between groups (HR, 1.54; 95% CI, 0.74-3.19; log-rank *P* = .245).

**Table IV:** Surgical failure/reoperation: 1-year and 2-year outcomes.

Cohort/timepoint	rTSA	ORIF	Risk difference	Risk ratio (95% CI)	HR (95% CI)	*P* value
65–74, 1 yr	18 (4.81%)	12 (3.21%)	+1.60%	1.50 (0.73-3.07)	1.54 (0.74-3.19)	.264
65–74, 2 yr	21 (5.62%)	13 (3.48%)	+2.14%	1.62 (0.82-3.18)	1.66 (0.83-3.31)	.160
75–84, 1 yr	19 (4.50%)	14 (3.32%)	+1.19%	1.36 (0.69-2.67)	1.37 (0.69-2.73)	.375
75–84, 2 yr	20 (4.54%)	25 (5.67%)	−1.13%	0.80 (0.45-1.42)	0.80 (0.45-1.45)	.444

CI, confidence interval; *ORIF*, open reduction and internal fixation; *rTSA*, reverse total shoulder arthroplasty; *HR*, hazard ratio (from Cox proportional hazards regression).

All comparisons were non-significant (*P* > .05).

In the 75 to 84-year cohort, 1-year surgical failure occurred in 19 rTSA patients (4.50%) and 14 ORIF patients (3.32%) (RR, 1.36; 95% CI, 0.69-2.67; *P* = .375). Time-to-event analysis likewise demonstrated no significant difference between treatments (HR, 1.37; 95% CI, 0.69-2.73; log-rank *P* = .369).

#### Two-year outcomes

At 2-year follow-up, no significant between-group difference in reoperation risk was observed in either age stratum. In the 65 to 74-year cohort, surgical failure occurred in 21 rTSA patients (5.62%) and 13 ORIF patients (3.48%) (RR, 1.62; 95% CI, 0.82-3.18; *P* = .160). Kaplan-Meier analysis remained non-significant (HR, 1.66; 95% CI, 0.83-3.31; log-rank *P* = .148).

In the 75 to 84-year cohort, 20 rTSA patients (4.54%) and 25 ORIF patients (5.67%) underwent reoperation by 2 years (RR, 0.80; 95% CI, 0.45-1.42; *P* = .444). Time-to-event analysis again showed no significant difference (HR, 0.80; 95% CI, 0.45-1.45; log-rank *P* = .466).

**Figure 4:**
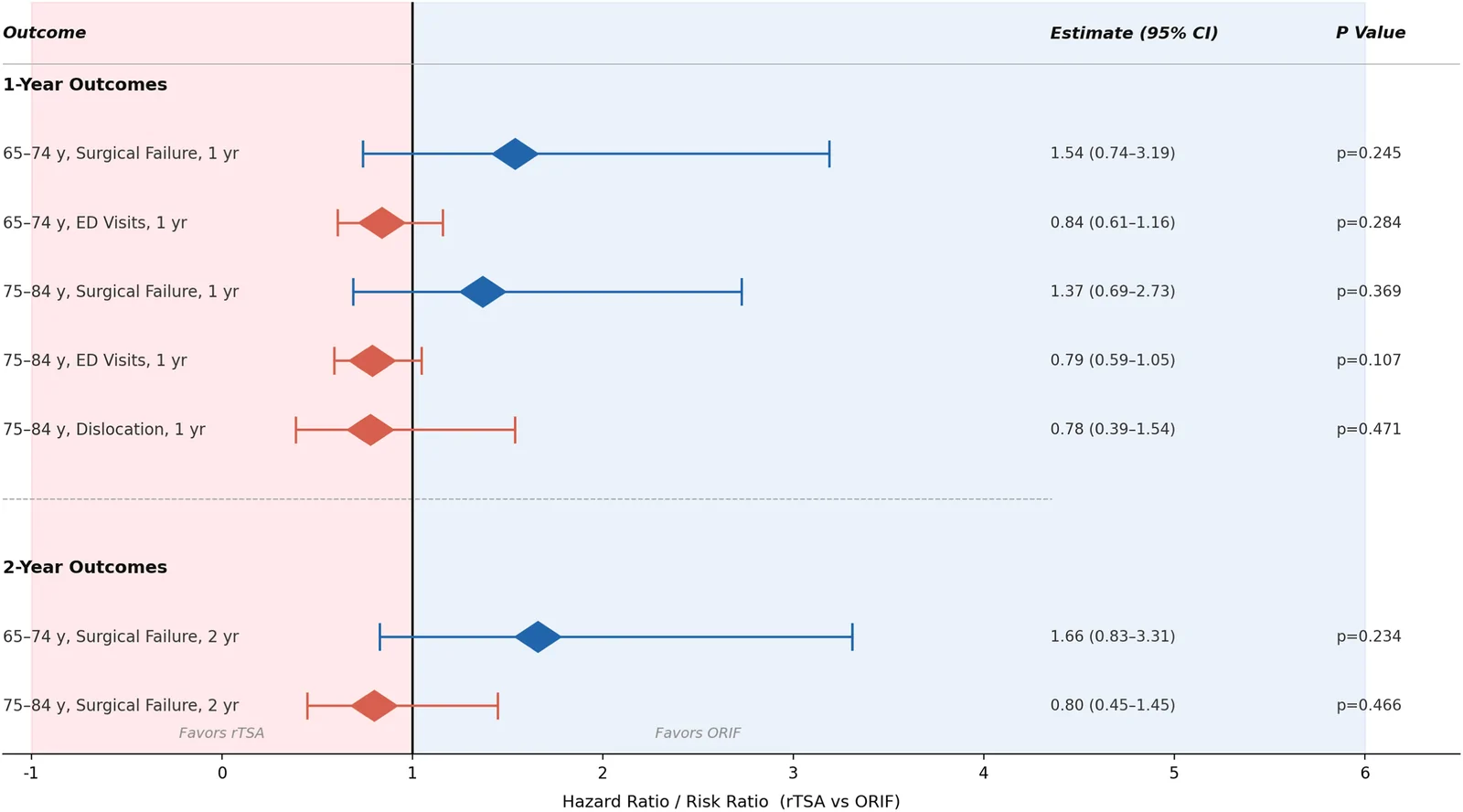
Forest plot of clinical outcomes: rTSA vs. ORIF by age cohort. Hazard ratios (surgical failure, Cox regression) and risk ratios (ED visits, dislocation) with 95% confidence intervals at 1- and 2-yr follow-up. Blue = 65-74 yr cohort; red = 75-84 yr cohort. Reference line at 1.0; values less than 1.0 indicate *Lower* event risk with rTSA, whereas values greater than 1.0 indicate *Lower* event risk with ORIF. All comparisons were non-significant (*P* > .05). *rTSA*, reverse total shoulder arthroplasty; *HR*, hazard ratio; *RR*, risk ratio; *CI*, confidence interval; *ED*, emergency department; *ORIF*, open reduction and internal fixation.

**Figure 5:**
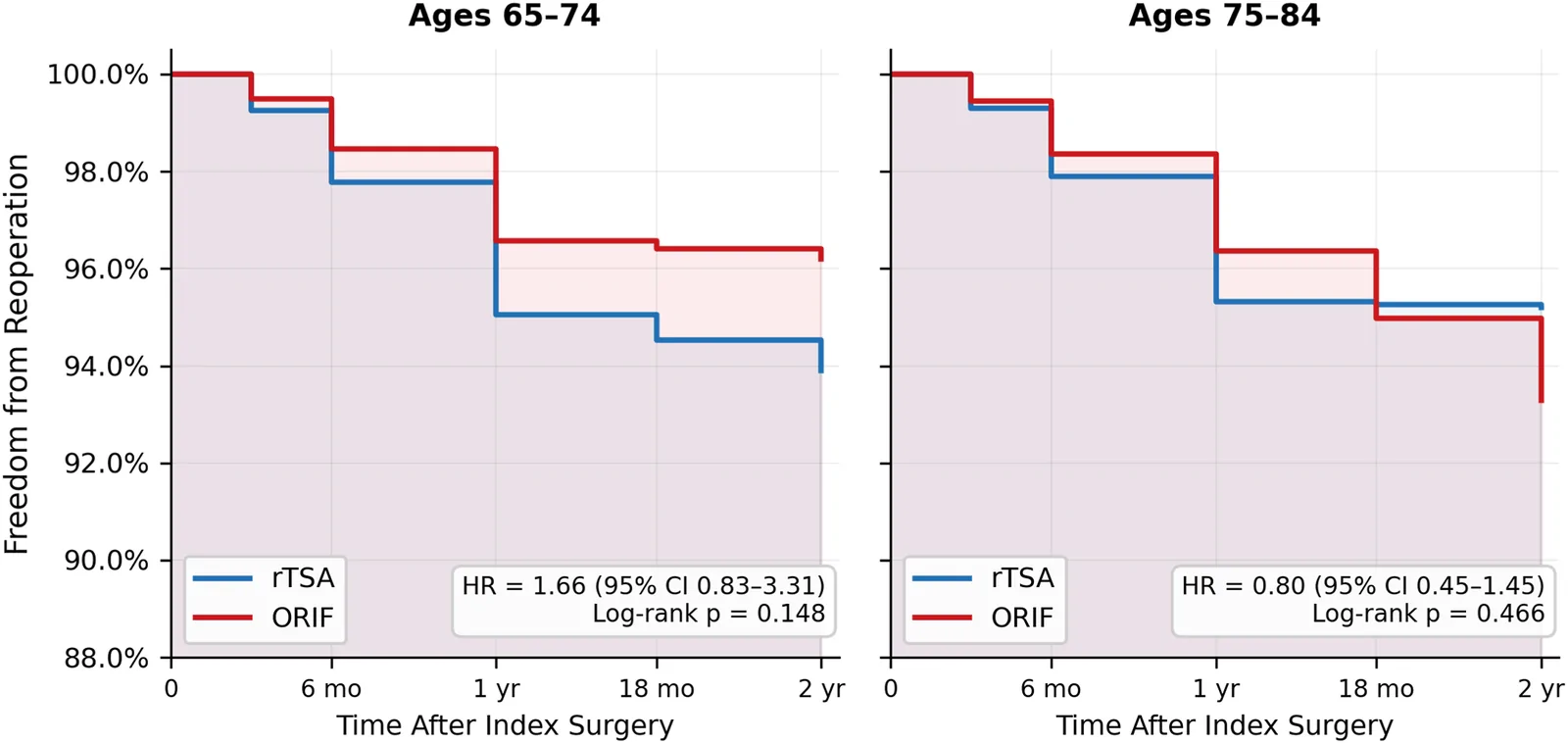
Kaplan–Meier survival analysis: freedom from surgical failure. Kaplan–Meier curves for freedom from reoperation in rTSA vs. ORIF at up to 2 yr, shown separately for each age cohort. Inset values reflect Cox proportional hazards regression. No significant difference was observed in either cohort (log-rank *P* > .05). *HR*, hazard ratio; *CI*, confidence interval; *rTSA*, reverse total shoulder arthroplasty; *ORIF*, open reduction and internal fixation.

**Figure 6:**
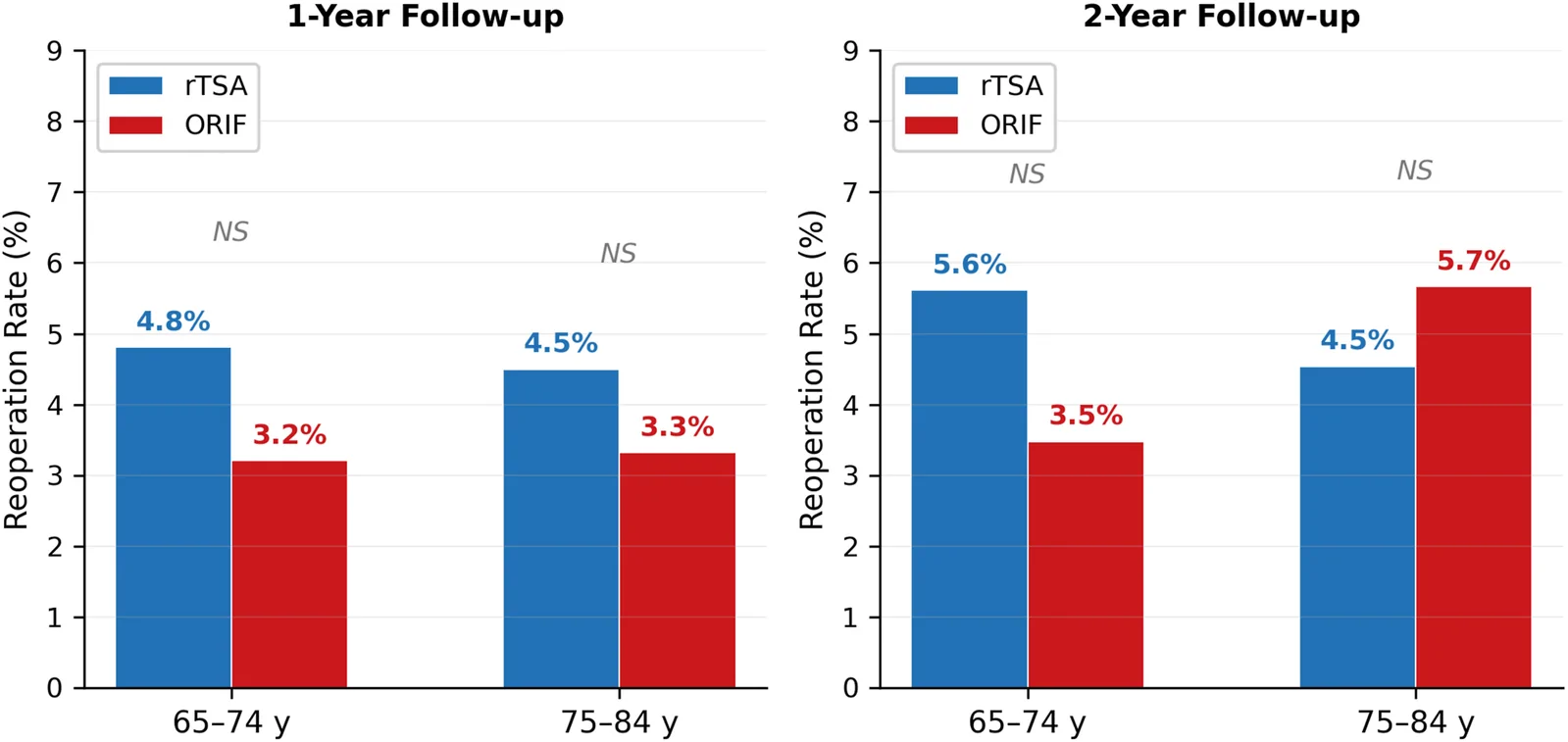
Reoperation rates by age cohort and follow-up duration. Crude reoperation rates for rTSA and ORIF at 1-yr (*Left*) and 2-yr (*Right*) follow-up by age cohort. No between-group difference was significant at either time point. In the 75-84 yr cohort, the 2-yr rTSA reoperation rate was numerically *Lower* than ORIF but not statistically significant (4.5% vs. 5.7%). *NS*, non-significant (*P* > .05); *rTSA*, reverse total shoulder arthroplasty; *ORIF*, open reduction and internal fixation.

Although these differences were not statistically significant, point estimates differed by age. In the younger elderly cohort, point estimates modestly favored ORIF, whereas in the older cohort, point estimates numerically favored rTSA (Fig. 4; Fig. 5; Fig. 6). These findings should be interpreted descriptively and not as evidence of a statistically significant treatment advantage.

### Secondary outcomes

At 1 year, ED utilization did not differ significantly between treatment groups in either age cohort (Table V). In the 65 to 74-year cohort, ED visits occurred in 58 rTSA patients (15.5%) and 69 ORIF patients (18.4%) (RR, 0.84; 95% CI, 0.61-1.16; *P* = .284). In the 75 to 84-year cohort, ED visits occurred in 67 rTSA patients (15.9%) and 85 ORIF patients (20.1%) (RR, 0.79; 95% CI, 0.59-1.05; *P* = .107). In the older cohort, Kaplan-Meier analysis suggested a non-significant trend toward lower ED utilization after rTSA (HR, 0.76; 95% CI, 0.55-1.04; log-rank *P* = .088).

**Table V:** Secondary outcomes at 1-year follow-up.

Outcome/cohort	rTSA	ORIF	Risk ratio (95% CI)	Odds ratio (95% CI)	*P* value
ED visit, 65–74	58 (15.5%)	69 (18.4%)	0.84 (0.61-1.16)	0.81 (0.55-1.19)	.284
ED visit, 75–84	67 (15.9%)	85 (20.1%)	0.79 (0.59-1.05)	0.75 (0.53-1.07)	.107
Dislocation, 75–84	14 (3.32%)	18 (4.27%)	0.78 (0.39-1.54)	0.77 (0.38-1.57)	.471

CI, confidence interval; *ORIF*, open reduction and internal fixation; *rTSA*, reverse total shoulder arthroplasty; *ED*, emergency department.

All comparisons were non-significant.

Dislocation data were available for the 75 to 84-year cohort at 1 year. Dislocation occurred in 14 rTSA patients (3.32%) and 18 ORIF patients (4.27%), with no significant difference between treatment groups (RR, 0.78; 95% CI, 0.39-1.54; odds ratio, 0.77; 95% CI, 0.38-1.57; *P* = .471). In the setting of administrative coding, this end point may capture a spectrum of instability or construct-related failure diagnoses rather than manually adjudicated glenohumeral dislocation events.

### Exploratory within-treatment age comparisons

Exploratory analyses were performed to compare younger and older patients within each treatment modality (Table VI). Among patients treated with rTSA, the 2-year reoperation rate was 5.97% in patients aged 65 to 74 years and 4.83% in those aged 75 to 84 years (RR, 1.24; 95% CI, 0.66-2.30; HR, 1.25; 95% CI, 0.66-2.37; *P* = .505). Among patients treated with ORIF, the corresponding reoperation rates were 3.48% and 5.22%, respectively (RR, 0.67; 95% CI, 0.37-1.20; HR, 0.67; 95% CI, 0.37-1.21; *P* = .170).

**Table VI:** Sub-group analysis: younger vs. older patients within treatment groups (2-year outcomes).

Treatment group	65–74 yr	75–84 yr	Risk ratio (95% CI)	HR (95% CI)	*P* value
rTSA	21/352 (5.97%)	17/352 (4.83%)	1.24 (0.66-2.30)	1.25 (0.66-2.37)	.505
ORIF	18/517 (3.48%)	27/517 (5.22%)	0.67 (0.37-1.20)	0.67 (0.37-1.21)	.170

CI, confidence interval; *HR*, hazard ratio; *ORIF*, open reduction and internal fixation; *rTSA*, reverse total shoulder arthroplasty.

Analysis compares younger (65–74 yr) vs. older (75–84 yr) patients within each treatment modality.

These sub-group comparisons were not statistically significant. Point estimates suggested relative stability of coded rTSA reoperation rates across age strata and numerically higher ORIF reoperation rates in the older age group, but these findings should be interpreted descriptively.

## Discussion

### Principal findings

In this age-stratified propensity score–matched analysis, rTSA and ORIF demonstrated comparable rates of coded reoperation at both 1 and 2 years in patients aged 65 to 74 years and 75 to 84 years. ED utilization was likewise similar between treatment groups, and dislocation was similar in the older cohort for which dislocation data were available. The most important interpretation is not treatment superiority, but rather equivalence of coded durability end points in a real-world database despite marked differences in fracture morphology. Because fracture pattern, patient-reported outcomes, bone quality, and surgical decision-making could not be fully captured, these findings should be considered associative and hypothesis-generating.^4^^,^^16^^,^^20^

### Age-stratified treatment effects

The numerical pattern observed in the 75 to 84-year cohort is consistent with the broader modern literature supporting arthroplasty in older patients with complex fractures, but it should not be interpreted as a statistically significant advantage in the present study. Fraser and colleagues reported superior outcomes for rTSA compared with plate fixation at 2 years in the DelPhi trial, and that superiority persisted at 5-year follow-up. Likewise, Lanzetti et al found that in patients older than 70 years with 3-part and 4-part fractures, rTSA produced better patient-reported outcomes and range of motion than angular stable plate fixation. Taken together, these studies provide clinical context for why surgeons may select rTSA more often in older patients with complex fractures, while the present administrative analysis demonstrates only comparable coded reoperation rates.^5^^,^^6^^,^^15^

At the same time, the absence of a clear rTSA advantage in the 65 to 74-year cohort suggests that ORIF likely retains an important role in selected younger elderly patients. This is biologically plausible. Patients in their late sixties and early seventies often represent a more heterogeneous group with variable bone quality, higher functional expectations, and, in some cases, fracture patterns that remain reconstructable. Prior comparative studies have shown that although rTSA may reduce revision burden, ORIF can preserve greater rotational function in selected patients, which may matter in a relatively younger, more active elderly population.^12^^,^^17^

### Fracture complexity and real-world treatment selection

One of the most important findings in this study was the persistent imbalance in fracture morphology between groups despite otherwise excellent postmatch covariate balance. Across both age strata, rTSA patients had markedly higher proportions of 4-part fractures, whereas ORIF patients had more 2-part fractures. This pattern mirrors real-world decision-making; however, it is also an important source of residual confounding because coded fracture morphology was not included in the matching algorithm. Contemporary treatment algorithms and comparative fracture studies consistently support arthroplasty for more complex, comminuted, and biologically compromised injuries, particularly when the risk of fixation failure is high.^5^^,^^7^^,^^19^

That context is essential when interpreting the primary findings. Because fracture complexity was not forced into the matching algorithm, the study preserved the clinical reality that surgeons preferentially choose rTSA for more severe fracture patterns. At the same time, this design prevents a definitive comparison of treatment effect matched by fracture pattern. Therefore, similar reoperation rates in the setting of greater coded fracture complexity among rTSA patients should be interpreted cautiously as a real-world association rather than proof that one treatment is superior to the other.^8^^,^^13^^,^^19^

### Reoperation risk must be interpreted alongside functional tradeoffs

Our results also highlight an important distinction between revision-based end points and functional end points. Several modern studies suggest that rTSA may outperform ORIF with respect to revision burden, but that does not necessarily mean it is uniformly superior across all clinically relevant domains. Klug et al reported fewer complications and fewer revisions with rTSA, yet also noted that some functional measures favored ORIF. Luciani et al similarly observed lower revision rates with rTSA despite worse rotational outcomes, while Lanzetti et al found better patient-reported outcomes and range of motion with rTSA in older patients. Meanwhile, the most recent systematic reviews continue to favor rTSA overall, especially for revision risk, but also acknowledge persistent heterogeneity in study design, fracture selection, and measured outcomes.^8^^,^^12^^,^^13^^,^^15^^,^^17^

This helps explain why our study, which centered on surgical failure/reoperation rather than function, did not reproduce a dramatic overall separation between treatment groups. A patient may avoid reoperation after either procedure yet experience meaningfully different motion, strength, or satisfaction. Accordingly, our data are best interpreted as a statement about comparative durability and health-care utilization, not as a definitive hierarchy of functional recovery.

### Early complications, instability, and health-care utilization

Another important nuance is that rTSA may not dominate ORIF across every phase of care. Köppe et al showed that in-hospital complications were more likely after rTSA than after locked plating, suggesting that perioperative burden may differ from longer-term revision burden. This distinction is relevant to the present study because a treatment can reduce late failure without necessarily reducing all early adverse events.^14^

In the present analysis, coded dislocation rates were low and comparable in the 75 to 84-year cohort for which dislocation data were available. This finding is reassuring but should be interpreted carefully because administrative diagnosis codes cannot distinguish true glenohumeral dislocation requiring reduction from subluxation, structural collapse, superior migration, or other instability-related coding. Contemporary fracture-specific arthroplasty studies have reported generally acceptable instability rates, and both Jain et al and Gallinet et al emphasized the importance of tuberosity healing and repair in optimizing function and potentially reducing complications after rTSA. These technical factors are not captured in database studies, but they likely influence outcomes in practice.^7^^,^^9^

All-cause ED utilization was numerically lower among older rTSA patients but did not reach statistical significance. Because ED visits were not limited to shoulder-specific presentations, this end point is best interpreted as a general health-care utilization marker. The direction of this non-significant finding is compatible with recent national data showing lower readmission rates after rTSA than after other treatment pathways for proximal humerus fractures and with economic analyses suggesting that rTSA can be cost-effective in older adults despite higher upfront implant costs.^1^^,^^11^

### Clinical implications

From a practical standpoint, these findings are consistent with an age-sensitive approach to surgical decision-making, but they should not be used as stand-alone evidence to select one operation over the other. ORIF remains a reasonable option for selected patients aged 65 to 74 years, particularly when fracture anatomy is reconstructable, bone quality is acceptable, and preservation of the native joint is desirable. For patients aged 75 to 84 years with complex 3-part or 4-part fractures, osteoporotic bone, or limited capacity to tolerate fixation failure, rTSA may remain an appropriate option; however, this study supports only comparable coded durability and health-care utilization outcomes, not functional superiority.^1^^,^^6^^,^^8^^,^^15^^,^^19^

Although prior literature has compared primary versus salvage rTSA after failed fixation, the present analysis did not directly evaluate salvage arthroplasty, revision indications, or functional outcomes after staged conversion. Therefore, conclusions regarding primary versus salvage rTSA are beyond the scope of these data and should be addressed by studies designed specifically for that question.^18^

### Limitations

This study has several limitations. First, its retrospective database design limits causal inference and remains subject to residual confounding despite propensity score matching. propensity scored matching can balance measured covariates but cannot account for unmeasured or inaccurately coded variables such as bone quality, rotator cuff status, baseline cognitive or functional status, activity level, surgeon preference, implant configuration, tuberosity repair, rehabilitation adherence, or radiographic fracture severity. Second, outcomes relied on administrative coding, which introduces potential misclassification of diagnoses, procedures, fracture morphology, ED utilization, and dislocation. Coded dislocation events may represent a heterogeneous group of instability, subluxation, collapse, or construct-failure diagnoses rather than classic glenohumeral dislocation. Third, fracture pattern was intentionally not included in the matching model, and failures could not be reliably stratified by 2-part, 3-part, and 4-part fracture morphology using the available matched output. Therefore, the greater proportion of 4-part fractures in the rTSA cohort remains an important source of residual confounding. Fourth, TriNetX does not capture important clinical details such as radiographic parameters, surgeon decision-making, implant-specific factors, operative notes, functional outcomes, or patient-reported measures. Fifth, ED utilization was analyzed as all-cause health-care use and should not be interpreted as a shoulder-specific complication. Finally, the definition of surgical failure as coded return to surgery may group together minor secondary procedures and major revision operations. Because multiple comparisons were performed without formal multiplicity correction, statistically non-significant findings and numerical differences should be interpreted cautiously and considered hypothesis-generating.

## Conclusion

rTSA and ORIF demonstrated comparable short-term coded durability in matched elderly patients with proximal humerus fractures. Despite being used in more complex coded fracture patterns, rTSA showed similar reoperation rates and all-cause ED utilization, with no statistically significant treatment advantage in either age stratum. These findings should be interpreted as hypothesis-generating real-world associations and highlight the need for prospective studies incorporating fracture-specific morphology, radiographic findings, and patient-reported functional outcomes.

## Disclaimers:

Funding: No funding was disclosed by the authors.

Conflicts of interest: The author, their immediate family, and any research foundation with which they are affiliated have not received any financial payments or other benefits from any commercial entity related to the subject of this article.

## Declaration of generative AI and AI-assisted technologies in the writing process

During the preparation of this work, the authors used ChatGPT (OpenAI) to assist with phrasing, organization, and grammar refinement. After using this tool, the authors reviewed, edited, and verified all content as needed and take full responsibility for the content of the manuscript.
